# Lipid stability, antioxidant potential and fatty acid composition of broilers breast meat as influenced by quercetin in combination with α-tocopherol enriched diets

**DOI:** 10.1186/s12944-015-0058-6

**Published:** 2015-06-24

**Authors:** Muhammad Sohaib, Masood Sadiq Butt, Muhammad Asim Shabbir, Muhammad Shahid

**Affiliations:** National Institute of Food Science and Technology, University of Agriculture, Faisalabad, Pakistan; Department of Biochemistry, University of Agriculture, Faisalabad, Pakistan

**Keywords:** Quercetin, Alpha tocopherol, TBARS, Fatty acids, MDA compounds

## Abstract

**Background:**

Dietary supplementation of antioxidants is a vital route to affect the oxidative stability and fatty acid profile of broiler meat. The supplementation of feed with antioxidants decreases degradation of lipids in muscles thereby enhances meat stability.

**Methods:**

The present study was carried out to investigate the influence of dietary quercetin in combination with α-tocopherol on growth performance, antioxidant potential, lipid stability and fatty acid composition in breast meat of birds. Accordingly, one day old 300 Hubbard strain male broiler birds were given three levels of quercetin @100, 200 and 300 mg/kg feed in combination with α-tocopherol @150, 225 and 300 mg/kg feed. The resultant meat was subjected to antioxidant assay, lipid stability, quantification of antioxidants followed by fatty acid profile of broiler breast meat.

**Results:**

The results explicated that feed treatments imparted momentous effect on gain in weight, and feed conversion efficiency however, intake of feed in birds affected non-momentously. The highest weight gain recorded in T9 as 2374.67 & 2388 g/bird followed by T8 & T6 2350 & 2353.33 and 2293.33 & 2307 g/bird, respectively whilst the lowest in T0 as 1992.67 & 1999 g/bird during the experimental year 2013 and 2014. The results regarding antioxidant potential revealed that among treatments, T9 exhibited highest values for total phenolic contents (TPC), 2,2-diphenyl-1-picrylhydrazyl (DPPH) & ferric reducing antioxidant power assay (FRAP) i.e. 158.70 ± 0.84 mg GAE/100 g, 82.40 ± 0.93 % and 682 ± 2.11 μmol/Fe^+2/^g, respectively as compared to T0 104.27 ± 1.64 mg GAE/100 g, 54.71 ± 0.64 % and 542.67 ± 1.74 μmol/Fe^+2^ /g of meat, correspondingly. The TBARS assay indicated that malondialdehydes production in meat increased during storage however, antioxidants deposition varied significantly among treatments. Fatty acid compositional analysis revealed that addition of quercetin with α-tocopherol in the bird’s diet decreased the fatty acid generation particularly saturated fatty acids.

**Conclusion:**

Conclusively, dietary supplementation of quercetin along with α-tocopherol improves growth performance, antioxidant capacity, stability of lipids and fatty acid composition in breast meat of birds.

## Introduction

Poultry meat is among the most popular meats in the world owing to its low price, short production time and ease of preparation [1]. The consumption of poultry meat is gaining popularity being an excellent source of high quality protein, possesses balanced amount of fatty acids mainly polyunsaturated fatty acids (PUFA) as well as provides essential vitamins and minerals. This meat contains higher levels of polyunsaturated fatty acids (PUFA) that trigger oxidative deterioration, resultantly decrease the quality of meat products [2]. The lipid oxidation is one of the major route for quality degradation in meat, apart from microbial spoilage. The mechanistic approach involves generation of reactive oxygen species and formation of free radicals, which produce rancid odor, off-flavor and surface discoloration of meat and meat products. The rate of lipid oxidation in fresh and cooked meat products depends on various internal factors such as fat content, fatty acid composition, level of antioxidants, heme pigment and iron contents. The end-products of this process impair color, aroma, flavor and texture of meat and allied products, hence reduce the nutritive value [3].

Oxidation problems in poultry meat and meat based products can be controlled through antioxidants such as synthetic ones like butylated hydroxyanisole (BHA), butylated hydroxytoluene (BHT), tert-butylhydroquinone (TBHQ) and propyl gallate (PG). However, theses synthetic counterparts are consider harmful for human health as well as controlled by regulatory agencies therefore, meat industry is looking for replacements of these synthetic antioxidants [4]. The quercetin [2-(3,4-dihydroxyphenyl)-3,5,7-trihydroxy- 4H-chromen-4-one] is one of flavonol belongs to class of flavonoids, ubiquitously present in fruits and vegetables specially red onion, caper, apple and in some medicinal & aromatic plants [5]. Several in vitro and in vivo studies conducted on human and experimental animals have revealed that quercetin possesses antioxidant and anti-inflammatory prospectives. The antioxidant activity of quercetin is attributed to its ability to scavenge free radicals, donate hydrogen atoms or electrons or chelate metal cations [6]. The pharmacokinetics of quercetin have proven that it ameliorates the absorption and metabolism of nutrients when enter the physiological system. The quercetin and its glycosidic metabolites possess antioxidant properties and modulate biological processes such as cell signaling pathways and reduction of oxidative DNA damage [7]. The oxidative deterioration of low density lipoproteins (LDL) can be prevented by dieatary quercetin as it possesses ability to scavenge free radicals and chelate transition metal ions generated through the oxidation of lipids. The α-tocopherol is the natural antioxidant that protects cells and tissues from oxidative damage induced by free radicals [8]. Several studies have reported that α-tocopherol is not deposited to a toxic level unlike other fat soluble antioxidants. Its accumulation at tissue level is strongly regulated through increasing hepatic metabolism that also regulates environmental toxins and speed of drug metabolism [9]. The supplementation of α-tocopherol in poultry meat been found to be one of pragmatic choice to improve oxidative stability of meat lipids and inhibiting oxidation of cholesterol. The inclusion of α-tocopherol in feed protects the broiler birds against stress-induced increase in thiobarbituric acid reactive substances (TBARS) by limiting oxidation as well as preserving animal health. It also enhances the lipid stability and quality of Beijing-you chicken muscles when supplemented through diet [10]. The supplementation of α-tocopherol at a level of 200 IU is considered effective to increase the oxidative stability of meat and its concentration in broiler muscles gradually raise in a dose dependent manner. Alpha tocopherol supplementation through feed improve the oxidative stability and quality of poultry meat and meat based products [11]. The objective of instant exploration was to examine the effect of dietary quercetin and α-tocopherol on growth performance, lipid stability, antioxidative potentialand fatty acid composition of broiler breast meat.

## Results and discussion

### Growth performance of birds

The results (Table 1) revealed that weight gain of broilers varied significantly among treatments however, non-significant behavior was noticed for experimental years. The highest weight gain is reported in T9 (2374.67 ± 3.53 & 2388 ± 6.43) followed by T8 and T6 as 2350 ± 6.93 & 2353.33 ± 4.0, 2293.33 ± 3.48 & 2307 ± 4.36 whilst lowest in T0 (1992.67 ± 4.37 & 1999 ± 6.81) g/bird, respectively in the year 2013 and 2014. The results elucidated maximum weight gain in broilers fed on higher level of dietary quercetin and α-tocopherol as compared to other treatments and control. Similarly, feed intake of birds ranged during 1st week ranged from 157.67 ± 3.38 to 176.33 ± 2.33 and 153.00 ± 11.4 to 165.33 ± 1.20 g/bird during the year 2013 and 2014, respectively while the values for this trait during 6th week of growth period varied from 976.00 ± 2.00 to 1035.33 ± 10.5 and 977.00 ± 1.53 to 1036.00 ± 10.4 g/bird for respective years. Nevertheless, feed intake of birds increased with progression of growth period and means during different weeks W1, W2, and W3, W4, W5, and W6 were 161.75 ± 0.90, 377.72 ± 0.69, 543.1 ± 0.73, 763.42 ± 1.22, 932.55 ± 1.38 and 1006.13 ± 2.79, correspondingly (Table 2). It is evident from results in Table 3 that feed conversion ratio (FCR) of birds varied significantly among treatments and weeks. The means indicated that birds fed on combinations of α-tocopherol and quercetin enriched diet reported lowest FCR in T9 as 1.686 ± 0.035 followed by T8 and T6 as 1.701 ± 0.031 & 1.722 ± 0.027 whilst, highest in T0 group (1.938 ± 0.026), respectively.

**Table 1:** Gain in weight of broiler birds fed on quercetin along with α-tocopherol supplemented feed

Treatments	Year	Weight gain (g/bird/week)						
		W_1_	W_2_	W_3_	W_4_	W_5_	W_6_	Means
T0	2013	133.3 ± 2.33	349.6 ± 6.06	638.3 ± 2.19	1049.4 ± 5.24	1545.6 ± 4.70	1992.7 ± 4.37	952.8 ± 111.04e
	2014	133.7 ± 1.76	349.0 ± 4.58	635.4 ± 4.06	1055.3 ± 4.70	1553.0 ± 3.61	1999.0 ± 6.81	
T1	2013	133.3 ± 1.20	343.3 ± 5.46	649.0 ± 3.46	1073.5 ± 1.86	1560.6 ± 2.91	2033.3 ± 4.98	966.6 ± 113.28de
	2014	136.7 ± 2.60	344.3 ± 2.85	646.3 ± 5.49	1071.0 ± 7.23	1569.7 ± 5.61	2039.0 ± 10.10	
T2	2013	135.6 ± 2.73	348.3 ± 7.97	656.6 ± 1.76	1106.6 ± 1.45	1607.7 ± 6.39	2111.7 ± 4.26	994.9 ± 117.67d
	2014	137.0 ± 1.53	348.0 ± 6.24	656.3 ± 7.84	1108.7 ± 4.67	1609.5 ± 8.76	2112.3 ± 6.69	
T3	2013	135.3 ± 1.20	348.2 ± 3.84	685.3 ± 3.18	1121.7 ± 4.98	1645.6 ± 5.30	2208.3 ± 6.01	1026.3 ± 122.99c
	2014	135.3 ± 1.76	348.3 ± 3.28	686 ± 5.29	1128.3 ± 4.06	1656.3 ± 7.31	2216.4 ± 7.54	
T4	2013	136 ± 2.08	348.6 ± 3.84	643.0 ± 1.53	1095.0 ± 2.65	1575.0 ± 5.57	2065.0 ± 7.55	986.2 ± 115.40 h
	2014	135.6 ± 2.03	349.0 ± 3.00	647.3 ± 6.96	1182.0 ± 6.43	1584.0 ± 7.57	2074.0 ± 6.24	
T5	2013	137.0 ± 3.51	351.3 ± 2.60	709.0 ± 1.15	1142.0 ± 4.73	1662.7 ± 5.93	2265.6 ± 7.75	1044.2 ± 125.47bc
	2014	139.0 ± 2.08	348.5 ± 1.45	706.4 ± 3.18	1136.3 ± 7.31	1661.6 ± 6.96	2271.0 ± 3.79	
T6	2013	137.7 ± 3.18	347.7 ± 6.33	730.0 ± 2.65	1152.7 ± 5.93	1679.0 ± 5.51	2293.3 ± 3.48	1057.5 ± 127.24b
	2014	137.3 ± 2.19	347.6 ± 4.33	724.6 ± 8.11	1154.3 ± 3.53	1679.6 ± 5.55	2307.0 ± 4.36	
T7	2013	135.6 ± 1.45	347.6 ± 4.98	669.7 ± 1.76	1102.0 ± 7.00	1628.3 ± 5.04	2157.0 ± 6.24	1007.6 ± 120.18 cd
	2014	135.0 ± 2.52	347.7 ± 3.48	669.6 ± 6.01	1108.3 ± 5.46	1627.0 ± 6.43	2163.3 ± 4.4	
T8	2013	134.7 ± 2.33	349.0 ± 2.65	745.7 ± 2.91	1164.3 ± 8.37	1695.0 ± 4.36	2350.0 ± 6.93	1073.6 ± 129.86ab
	2014	135.3 ± 2.91	349.3 ± 1.76	744.3 ± 5.81	1169.0 ± 6.66	1693.4 ± 5.21	2353.3 ± 4.06	
T9	2013	135.6 ± 0.88	348.6 ± 4.33	762.0 ± 4.62	1180.0 ± 2.31	1713.3 ± 2.40	2374.6 ± 3.53	1086.5 ± 131.45a
	2014	135.3 ± 3.18	349.0 ± 3.79	763.7 ± 6.94	1180.3 ± 4.98	1708.0 ± 5.29	2388.0 ± 6.43	
Means		135.7 ± 0.46f	348.2 ± 0.85e	688.4 ± 5.62d	1124.1 ± 5.40c	1632.8 ± 7.0b	2188.7 ± 16.74a	

Data are means ± SE. Means sharing similar letters in a column do not differ significantly from one another (p˃0.05)

T0 = control without antioxidants, T1 = 100 mg quercetin + 150 mg α-tocopherol/kg feed; T2 = 100 mg quercetin + 225 mg α-tocopherol/kg feed; T3 = 100 mg quercetin + 300 mg α-tocopherol/kg feed; T4 = 200 mg quercetin + 150 mg α-tocopherol/kg feed; T5 = 200 mg quercetin + 225 mg α-tocopherol/kg feed; T6 = 200 mg quercetin + 300 mg α-tocopherol/kg feed, T7 = 300 mg quercetin + 150 mg α-tocopherol/kg feed; T8 = 300 mg quercetin + 225 mg α-tocopherol/kg feed, T9 = 300 mg quercetin + 300 mg α-tocopherol/kg feed

**Table 2:** Feed intake of broiler birds

Treatments	Year	Feed intake (g/bird/week)						Means
		W_1_	W_2_	W_3_	W_4_	W_5_	W_6_	
T0	2013	161 ± 2.08	380 ± 2.60	538 ± 2.60	756 ± 5.71	950 ± 3.28	976 ± 2.00	628.08 ± 50.17
	2014	165 ± 1.20	381 ± 3.76	538 ± 3.18	759 ± 5.70	953 ± 3.71	977 ± 1.53	
T1	2013	176 ± 2.33	378 ± 2.89	542 ± 2.73	756 ± 8.95	919 ± 4.67	978 ± 1.20	624.14 ± 49.10
	2014	160 ± 2.31	377 ± 3.18	544 ± 3.28	758 ± 7.54	923 ± 4.67	979 ± 2.41	
T2	2013	162 ± 4.10	377 ± 2.65	543 ± 2.33	776 ± 9.28	931 ± 3.61	994 ± 4.00	631.14 ± 50.63
	2014	163 ± 2.73	373 ± 2.08	545 ± 2.60	778 ± 9.54	934 ± 3.46	998 ± 3.06	
T3	2013	158 ± 3.38	380 ± 2.31	538 ± 2.08	762 ± 3.67	933 ± 1.23	1007 ± 6.96	630.28 ± 51.07
	2014	157 ± 1.76	380 ± 0.88	538 ± 3.93	764 ± 3.84	936 ± 4.84	1009 ± 4.93	
T4	2013	160 ± 2.60	376 ± 3.06	544 ± 2.08	763 ± 4.26	930 ± 3.21	996 ± 3.180	629.14 ± 50.53
	2014	164 ± 2.03	372 ± 3.51	545 ± 2.08	767 ± 3.61	934 ± 4.16	999 ± 1.76	
T5	2013	162 ± 2.60	378 ± 3.21	542 ± 3.21	763 ± 4.33	933 ± 4.51	996 ± 3.46	630.03 ± 50.57
	2014	161 ± 2.33	378 ± 4.16	545 ± 2.03	765 ± 4.33	935 ± 3.28	1001 ± 2.03	
T6	2013	161 ± 2.91	376 ± 2.89	542 ± 2.31	763 ± 2.65	939 ± 4.33	1024 ± 14.53	634.61 ± 51.52
	2014	163 ± 3.61	377 ± 1.76	546 ± 3.93	766 ± 2.65	940 ± 2.85	1017 ± 6.57	
T7	2013	164 ± 6.11	378 ± 1.15	542 ± 3.53	759 ± 2.60	927 ± 2.40	1015 ± 4.67	630.39 ± 51.14
	2014	153 ± 11.40	378 ± 4.16	539 ± 4.06	761 ± 2.33	931 ± 3.53	1017 ± 3.79	
T8	2013	160 ± 3.46	374 ± 2.03	545 ± 2.08	765 ± 2.85	932 ± 3.21	1035 ± 10.55	636.31 ± 52.03
	2014	160 ± 2.96	377 ± 4.73	545 ± 1.86	768 ± 3.00	937 ± 2.45	1036 ± 10.46	
T9	2013	162 ± 1.73	383 ± 2.40	554 ± 5.03	757 ± 4.10	914 ± 4.16	1033 ± 4.81	633.67 ± 51.19
	2014	162 ± 2.03	379 ± 6.57	543 ± 2.65	760 ± 4.41	920 ± 2.31	1035 ± 3.71	
Means		162 ± 0.90f	378 ± 0.69e	543 ± 0.73d	763 ± 1.22c	932 ± 1.38b	1006 ± 2.79a	

Data are means ± SE. Means sharing similar letters in a column do not differ significantly from one another (p>0.05)

T0 = control without antioxidants, T1 = 100 mg quercetin + 150 mg α-tocopherol/kg feed; T2 = 100 mg quercetin + 225 mg α-tocopherol/kg feed; T3 = 100 mg quercetin + 300 mg α-tocopherol/kg feed; T4 = 200 mg quercetin + 150 mg α-tocopherol/kg feed; T5 = 200 mg quercetin + 225 mg α-tocopherol/kg feed; T6 = 200 mg quercetin + 300 mg α-tocopherol/kg feed, T7 = 300 mg quercetin + 150 mg α-tocopherol/kg feed; T8 = 300 mg quercetin + 225 mg α-tocopherol/kg feed, T9 = 300 mg quercetin + 300 mg α-tocopherol/kg feed

**Table 3:** Effect of feed treatments on feed conversion ratio of broiler birds

Treatments	Year	Feed conversion ratio						Means
		W_1_	W_2_	W_3_	W_4_	W_5_	W_6_	
T0	2013	2.10 ± 0.05	1.76 ± 0.05	1.86 ± 0.02	1.84 ± 0.02	1.91 ± 0.01	2.18 ± 0.02	1.94 ± 0.03a
	2014	2.02 ± 0.04	1.77 ± 0.02	1.87 ± 0.01	1.81 ± 0.03	1.92 ± 0.02	2.19 ± 0.04	
T1	2013	2.12 ± 0.04	1.80 ± 0.06	1.77 ± 0.02	1.78 ± 0.04	1.89 ± 0.01	2.07 ± 0.04	1.89 ± 0.03ab
	2014	1.89 ± 0.05	1.82 ± 0.03	1.80 ± 0.03	1.78 ± 0.02	1.85 ± 0.05	2.09 ± 0.06	
T2	2013	1.90 ± 0.07	1.78 ± 0.07	1.76 ± 0.05	1.73 ± 0.01	1.86 ± 0.01	1.97 ± 0.03	1.84 ± 0.02bc
	2014	1.917 ± 0.05	1.77 ± 0.06	1.77 ± 0.05	1.72 ± 0.05	1.87 ± 0.04	1.98 ± 0.01	
T3	2013	1.84 ± 0.01	1.783 ± 0.05	1.60 ± 0.02	1.75 ± 0.02	1.78 ± 0.03	1.79 ± 0.04	1.77 ± 0.02 cd
	2014	1.89 ± 0.02	1.787 ± 0.03	1.60 ± 0.04	1.73 ± 0.02	1.77 ± 0.04	1.81 ± 0.05	
T4	2013	1.85 ± 0.02	1.77 ± 0.04	1.85 ± 0.03	1.69 ± 0.01	1.94 ± 0.02	2.03 ± 0.01	1.87 ± 0.04b
	2014	1.96 ± 0.06	1.74 ± 0.02	1.83 ± 0.06	1.44 ± 0.04	2.33 ± 0.09	2.04 ± 0.03	
T5	2013	1.87 ± 0.01	1.77 ± 0.06	1.51 ± 0.01	1.76 ± 0.03	1.79 ± 0.03	1.65 ± 0.03	1.73 ± 0.02d
	2014	1.85 ± 0.02	1.80 ± 0.03	1.52 ± 0.01	1.79 ± 0.04	1.78 ± 0.01	1.64 ± 0.02	
T6	2013	1.85 ± 0.08	1.79 ± 0.06	1.42 ± 0.02	1.80 ± 0.03	1.78 ± 0.01	1.67 ± 0.02	1.72 ± 0.03d
	2014	1.91 ± 0.07	1.79 ± 0.05	1.45 ± 0.02	1.78 ± 0.02	1.78 ± 0.01	1.62 ± 0.01	
T7	2013	1.91 ± 0.08	1.79 ± 0.03	1.68 ± 0.02	1.75 ± 0.03	1.76 ± 0.01	1.92 ± 0.05	1.80 ± 0.02c
	2014	1.84 ± 0.08	1.78 ± 0.03	1.68 ± 0.06	1.73 ± 0.01	1.79 ± 0.02	1.90 ± 0.05	
T8	2013	1.88 ± 0.06	1.75 ± 0.02	1.37 ± 0.01	1.83 ± 0.04	1.75 ± 0.01	1.58 ± 0.01	1.70 ± 0.03de
	2014	1.93 ± 0.05	1.76 ± 0.04	1.38 ± 0.01	1.81 ± 0.04	1.78 ± 0.04	1.57 ± 0.02	
T9	2013	1.88 ± 0.03	1.80 ± 0.05	1.34 ± 0.01	1.81 ± 0.02	1.71 ± 0.01	1.56 ± 0.01	1.68 ± 0.04e
	2014	1.94 ± 0.07	1.78 ± 0.08	1.31 ± 0.02	1.82 ± 0.02	1.74 ± 0.04	1.53 ± 0.02	
Means		1.92 ± 0.02a	1.78 ± 0.01c	1.62 ± 0.02d	1.76 ± 0.02c	1.84 ± 0.02b	1.84 ± 0.03b	

Data are means ± SE. Means sharing similar letters in a column do not differ significantly from one another (p˃0.05)

T0 = control without antioxidants, T1 = 100 mg quercetin + 150 mg α-tocopherol/kg feed; T2 = 100 mg quercetin + 225 mg α-tocopherol/kg feed; T3 = 100 mg quercetin + 300 mg α-tocopherol/kg feed; T4 = 200 mg quercetin + 150 mg α-tocopherol/kg feed; T5 = 200 mg quercetin + 225 mg α-tocopherol/kg feed; T6 = 200 mg quercetin + 300 mg α-tocopherol/kg feed, T7 = 300 mg quercetin + 150 mg α-tocopherol/kg feed; T8 = 300 mg quercetin + 225 mg α-tocopherol/kg feed, T9 = 300 mg quercetin + 300 mg α-tocopherol/kg feed

The findings of instant study are supported by the findings of [12], noticed weight gain in broilers fed on α-tocopherol @200 mg/kg of feed. One of the researchers groups, [13] indicated that higher concentration of α-tocopherol in feed improved growth performance by yielding higher weight gain. Earlier, [14] stated that dietary supplementation of quercetin enhanced the weight gain and growth efficiency in broilers at a level of 200 ppm/kg via diet. Recently, [15] reported that dietary supplementation of quercetin at a concentration of 0.1 and 1 g/kg feed enhanced weight gain of chickens however, reprted non-significant effect for feed intake of birds. Similar findings were reported by [16], observed non-momentous effect of hesperidin (3 g/kg of feed) on feed intake of birds. One of scientists groups, [17] delineated that feeding α-tocopherol supplemented diet has non momentous impact on birds feed intake. The previous findings of [18] also indicated non substancial effect on feed intake by supplementation of α-tocopherol. Current results regarding FCR of broilers administrated quercetin and α-tocopherol supplemented feed are concordant with the work of [19], reported that inclusion of carvacrol and quercetin @ 200 mg/kg feed improved FCR of broilers. Likewise, [17] observed improved FCR of broiler birds linearly with increasing concentration of α-tocopherol. One of the researchers groups, [15] noticed lower feed conversion ratio (p < 0.05) when birds were provided feed containing higher level of quercetin. The results of previous findings of [20] also indicated that efficiency of feed was increased with α-tocopherol fortification in birds. In the current exploration, the average mortality rate for broilers fed on combination of dietary quercetin and alpha tocopherol enriched feed was ranged from 5–6.1 %, however, 7.3 % for control group birds.

### Antioxidant potential of breast meat

#### Total phenolic contents

The results indicated that total phenolic contents (TPC) in samples of birds varied significantly among treatments but differed non-significantly due to years (Table 4). Means reported that total phenolic contents of breast meat for different broiler groups T0, T1, T2, T3, T4, T5, T6, T7, T8 and T9 were 103.87 ± 0.94, 119.73 ± 1.04, 121.10 ± 1.82, 124.73 ± 0.49, 135.33 ± 1.58, 137.47 ± 1.30, 139.77 ± 1.35, 153.13 ± 0.61, 157.23 ± 0.55, 158.70 ± 0.84 (mg GAE/100 g meat) in 1st trial while the value of this trait for 2nd trial were 104.67 ± 3.52, 118.80 ± 1.62, 119.70 ± 0.67, 122.63 ± 1.60, 134.10 ± 0.55, 135.63 ± 0.43, 139.13 ± 0.61, 152.53 ± 1.22, 156.27 ± 0.50, 157.63 ± 0.54 (mg GAE/100 g meat), in respective treatments. The highest TPC were reported in T9 (158.17 ± 0.51) followed by T8 and T7 (156.75 ± 0.39 and 152.83 ± 0.63) whereas lowest in T0 (104.27 ± 1.64) at completion of the study. The results of instant study are supported by the findings of [21] who reported that antioxidants provided through diet are deposited in muscles thereby alleviate oxidative stress of animals by increasing concentration of phenolic compounds. They further stated that deposition of phenolic compounds is higher in breast muscles than that of leg meat of birds. Similarly, [22] indicated that broilers fed on α-tocopherol supplemented feed resulted higher antiradical power due to the accumulation of phenolic compounds. Likewise, [23] also showed that feed containing exogenous antioxidants like vitamin C & E, ubiquinols and polyphenols enhance total phenolic potential of poultry meat when birds are fed on antioxidant enriched feed. One of the researchers groups, [24] observed that total phenolic potential of pig meat was enhanced by increasing concentration of phenolic compounds through feed.

**Table 4:** Total phenolic contents and free radical scavenging activity in breast meat of birds

Treatments	TPC (mg GAE/100 g meat)			DPPH scavenging activity (%)		
	Year 1	Year 2	Mean	Year 1	Year 2	Mean
T0	103.87 ± 0.94	104.67 ± 3.52	104.27 ± 1.64f	54.21 ± 0.68	55.20 ± 1.15	54.71 ± 0.64f
T1	119.73 ± 1.04	118.80 ± 1.62	119.27 ± 0.88e	61.40 ± 0.83	59.90 ± 0.71	60.65 ± 0.59ef
T2	121.10 ± 1.82	119.70 ± 0.67	120.40 ± 0.92de	62.15 ± 0.90	61.47 ± 0.58	61.81 ± 0.50e
T3	124.73 ± 0.49	122.63 ± 1.60	123.68 ± 0.88 cd	67.39 ± 0.99	67.35 ± 0.95	67.37 ± 0.61d
T4	135.33 ± 1.58	134.10 ± 0.55	134.72 ± 0.80d	64.58 ± 1.60	62.75 ± 0.50	63.67 ± 0.85de
T5	137.47 ± 1.30	135.63 ± 0.43	136.55 ± 0.74c	71.58 ± 1.45	71.08 ± 1.28	71.33 ± 0.87c
T6	139.77 ± 1.35	139.13 ± 0.61	139.45 ± 0.68b	77.17 ± 1.17	77.26 ± 0.55	77.22 ± 0.58b
T7	153.13 ± 0.61	152.53 ± 1.22	152.83 ± 0.63bc	73.24 ± 1.01	72.92 ± 1.75	73.08 ± 0.91bc
T8	157.23 ± 0.55	156.27 ± 0.50	156.75 ± 0.39ab	81.55 ± 1.21	79.43 ± 0.72	80.49 ± 0.79ab
T9	158.70 ± 0.84	157.63 ± 0.54	158.17 ± 0.51a	82.95 ± 1.41	81.85 ± 1.44	82.40 ± 0.93a
Means	135.11 ± 3.18	134.11 ± 3.16		69.62 ± 1.67	68.92 ± 1.61	

Means sharing similar letters in a column do not differ significantly from one another (*p* 0.05)

T0 = control without antioxidants, T1 = 100 mg quercetin + 150 mg α-tocopherol/kg feed; T2 = 100 mg quercetin + 225 mg α-tocopherol/kg feed; T3 = 100 mg quercetin + 300 mg α-tocopherol/kg feed; T4 = 200 mg quercetin + 150 mg α-tocopherol/kg feed; T5 = 200 mg quercetin + 225 mg α-tocopherol/kg feed; T6 = 200 mg quercetin + 300 mg α-tocopherol/kg feed, T7 = 300 mg quercetin + 150 mg α-tocopherol/kg feed; T8 = 300 mg quercetin + 225 mg α-tocopherol/kg feed, T9 = 300 mg quercetin + 300 mg α-tocopherol/kg feed

#### Free radical scavenging activity by using DPPH Assay

The statistical analysis indicted that DPPH assay of broiler meat samples varied significantly among treatments (Table 4). In treatments, the lowest value in DPPH value of breast meat was noticed in T0 (control) 54.71 ± 0.64 % whereas, the highest in T9 (birds fed on 300 mg quercetin & 300 mg of α-tocpherol/kg feed) 82.40 ± 0.93 % followed by T8 (birds fed on 300 mg quercetin & 225 mg of α-tocpherol/kg feed) 80.49 ± 0.79 %, T6 (birds fed on 200 mg quercetin and 300 mg of α-tocpherol/kg feed) 77.22 ± 0.58 %, respectively. It is obvious from the results that free radical scavenging activity showed identical trend in study years however, the activity in meat increased linearly. The results of present study are in harmony with [25]), observed free radical scavenging activity of three flavonoids i.e. quercetin, kaempferol and pterostilbene. They noticed the highest antiradical ability of quercetin followed by kaempferol and pterostilbene. Likewise, [26] also indicated that α-tocopherol alone or in combination with red ginseng enhanced the DPPH radical scavenging capacity of meat. The mechanistic approach elaborates that antioxidants combine with free radicals, inactivate them thus decreases the free radicals intracellular concentration which enhances oxidative stability of meat. Similarly, [27] noticed that vitamin E and sage extract increased the antiradical power of poultry meat than that of control.

#### Ferric reducing antioxidant power

**Fig. 1:**
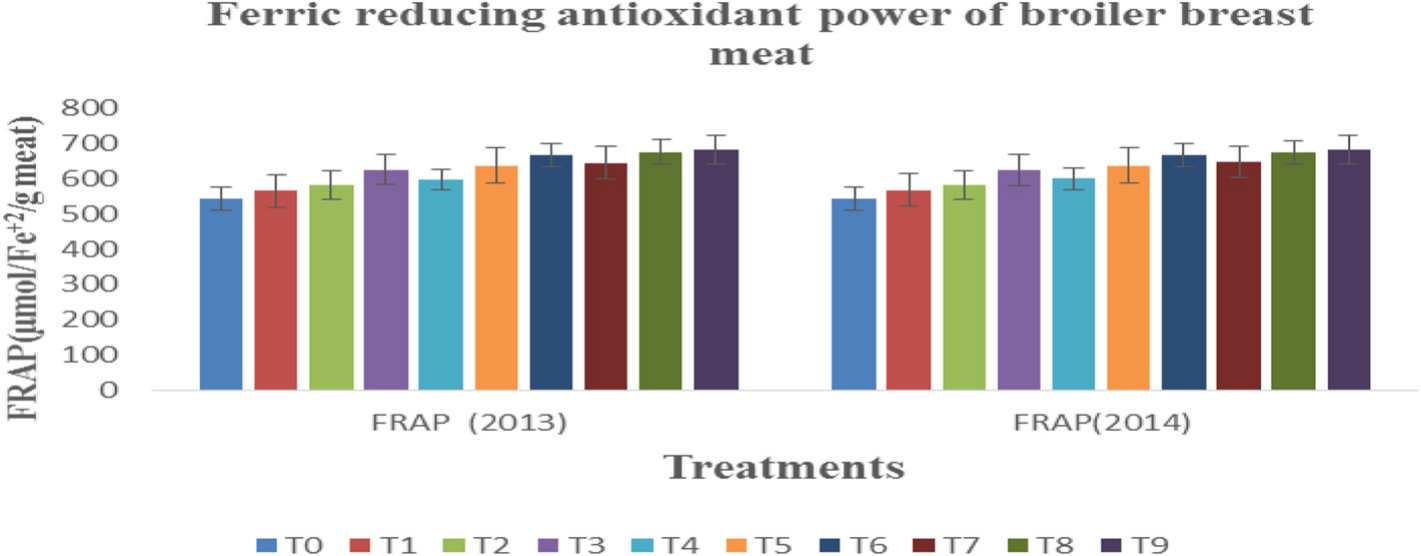
Ferric reducing antioxidant power of broiler breast meat for the year 2013 and 2014. T0 = control without antioxidants;T1 = 100 mg quercetin + 150 mg α-tocpherol/kg feed; T2 = 100 mg quercetin + 225 mg α-tocpherol/kg feed; T3 = 100 mg quercetin + 300 mg α-tocpherol/kg feed; T4 = 200 mg quercetin + 150 mg α-tocpherol/kg feed;T5 = 200 mg quercetin + 225 mg α-tocpherol/kg feed; T6 = 200 mg quercetin + 30 0 mg α-tocpherol/kg feed; T7 = 300 mg quercetin + 150 mg α-tocpherol/kg feed; T8 = 300 mg quercetin + 225 mg α-tocpherol/kg feed; T9 = 300 mg quercetin + 300 mg α-tocpherol/kg feed

Ferric reducing antioxidant power (FRAP) assay is an important indicator to estimate the antioxidant potential of different foods based on chelating capacity of ferrous ion (Fe^3+^ to Fe^2+^). The treatments imparted momentous effect on FRAP of breast meat of broilers however, years exhibited non-momentous effect. The results regarding FRAP of broiler meat in different groups depicted that amongst treatments, the minimum FRAP value for breast meat was in T0 as 543.67 ± 1.86 & 541.67 ± 3.28 μmol/Fe^+2^/g meat while, the maximum in T9, T8 and T6 as 683.00 ± 3.79 & 681.00 ± 2.65, 675.33 ± 3.48 & 674.67 ± 2.03, 666.00 ± 2.08& 667.00 ± 2.08 μmol/Fe^+2^/g meat, respectively in the year 2013 and 2014 (Fig. 1). The current findings are concordant with work of, [28] also found higher ferric reducing power in meat containing natural antioxidants compared to synthetic counterparts. They further elaborated that antioxidants in meat decrease the reductants in meat that are involved in conversion of Fe3+ to Fe2+. Similarly, [29] delineated that total antioxidant capacity of broiler meat as measured by FRAP is significantly varied when birds are provided feed enriched with 300 & 600 mg quercetin/kg body weight.

#### Oxidative stability by thiobarbituric acid reactive substances assay

**Fig. 2:**
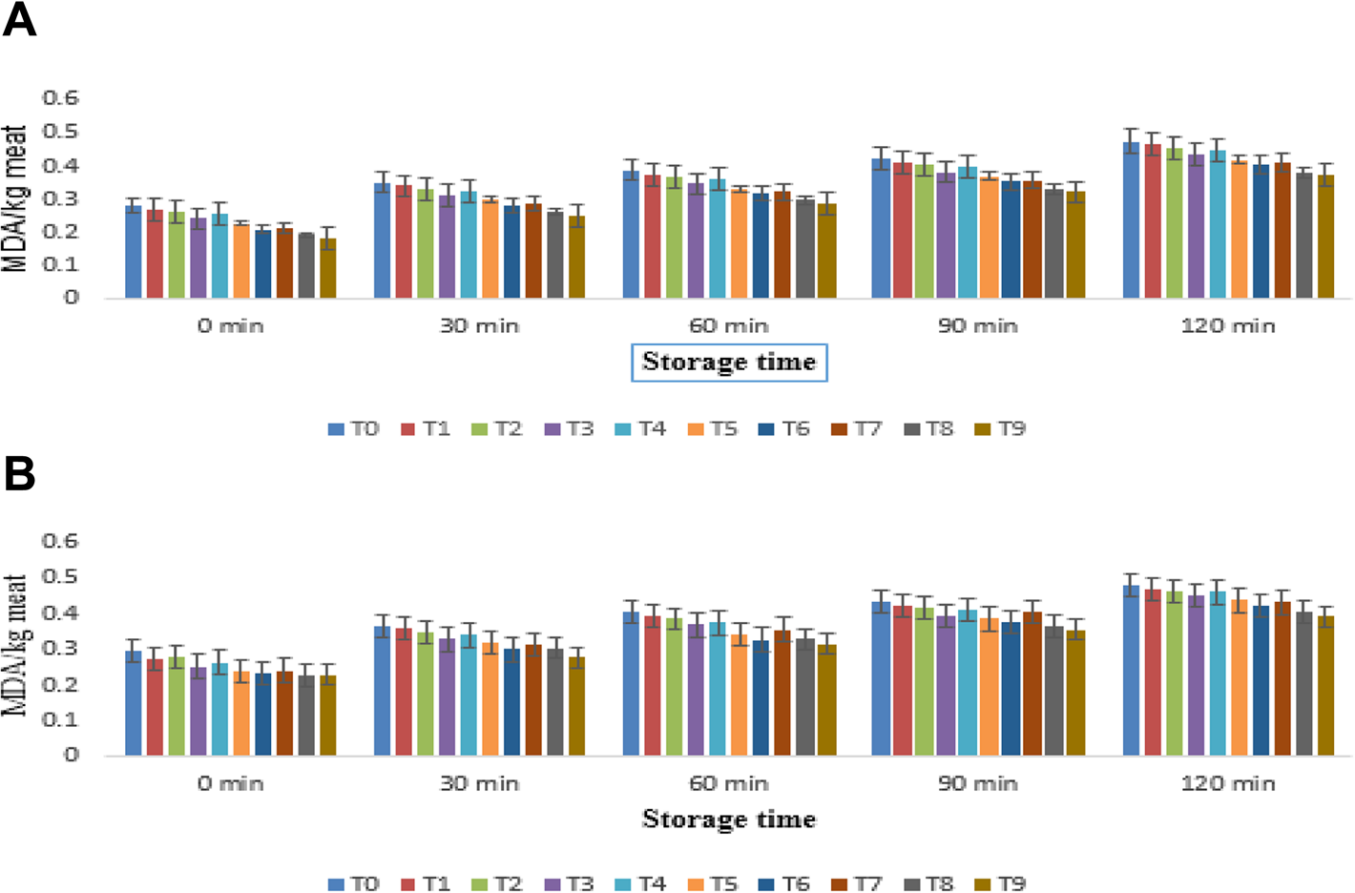
Thiobarbituric acid reactive substances (TBARS) of broiler breast meat fed on quercetin and α-tocopherol supplemented feed. **a**. TBARS for broiler breast meat fed on antioxidant enriched feed during the year 2013. **b**. TBARS for broiler breast meat fed on antioxidant enriched feed during the year 2013

The results regarding thiobarbituric acid reactive substances (TBARS) of breast meat of broiler showed significant differences due to treatments (Fig. 2). The means in Fig. 2 explicated that TBARS of breast meat on initiation (1st min) of storage varied from 0.181 ± 0.0032 to 0.282 ± 0.0023 and 0.229 ± 0.0153 to 0.441 ± 0.1594 mg of MDA/kg meat whereas at 120th min from 0.283 ± 0.0174 to 0.384 ± 0.0175 and 0.314 ± 0.0157 to 0.425 ± 0.0289 mg of MDA/kg meat among treatments during the year 2013 and 2014, respectively. The lowest TBARS of breast meat recorded in T9 as 0.298 ± 0.0119 mg of MDA/kg meat followed by T8 and T6 as 0.309 ± 0.0120 and 0.315 ± 0.0184 mg of MDA/kg meat whilst, maximum in T0 0.405 ± 0.0170 mg of MDA/ kg meat. Similarly, TBARS of leg meat in T9, T8, T6 and T0 were 0.305 ± 0.0130, 0.315 ± 0.0125, 0.332 ± 0.0128, 0.406 ± 0.0128 mg MDA/kg meat, respectively at the end of trial. It is evident from exploration that lower MDA production reported in breast meat compared to leg meat however, TBARS increased as a function of storage. Previous studies showed that quercetin and α-tocopherol have ability to attenuate the process of lipid peroxidation. It has been observed from findings of [15], noticed the impact of dietary quercetin @ 0.5 and 1 g/kg of feed to the birds. It has been inferred that oxidative stability of broiler meat under refrigerated as measured by TBARS was enhanced (p < 0.05) when birds were fed quercetin supplemented diet @ 1 g/kg feed. The TBARS value in different groups at initiation of storage were 24.3 ng of MDA/g meat (control), 23.3 ng of MDA/g meat (0.5 g/kg feed) and 20.2 ng of MDA/g meat (1 g/kg feed) that progressively increased to 95.5, 73.7 and 64.6 ng of MDA/g meat in respective treatments at termination. One of the researchers groups, [30] observed concomitant increase in lipid peroxidation in pig meat as indicated by higher (p < 0.05) concentrations of TBARS and 8-iso-PGF2 nevertheless, it was ameliorated (p < 0.05) by dietary fortification of quercetin. Likewise, [14] also found that supplementing poultry diet with quercetin @200 ppm/kg feed substantially reduced TBARS. Recently, [31] reported that quercetin alone or in combination with flaxseed reduces the formation of oxysterol in meat (p < 0.05), an oxidation product of cholesterol and lipids after 7 days of refrigerated storage. One of the researchers groups, [32] expounded that α-tocopherol alone or in combination with rosemary & green tea extract retards lipid oxidation in meat.

#### Quercetin contents

The results in Table 5 revealed momentous differences in quercetin content of antioxidant enriched broiler breast meat due to treatments. The highest quercetin were measured in T9 16.36 ± 1.01 mg/kg meat followed T8 and T3 as 14.67 ± 0.03 and 14.13 ± 0.06 mg/kg meat, respectively nonetheless, qurcetin was not recorded in T0 (control) group. The results of instant study are in harmony with [29], reported that quercetin and its metabolites such as quercetin glucuronides and sulfonates are deposited in liver, breast and thigh muscles when birds are provided quercetin supplemented feed. Likewise, [33] also found that quercetin level of turkey roll increased by the addition of onion juice brine (OJB) directly in meat @25 (OJB25) and 50 % (OJB50) solution. They further reported quercetin content of turkey rolls 8.17 and 16.3 mg/kg meat in 25 and 50 % brine solution, respectively. However, cooking process depleted quercetin in resultant meat after cooking (30 min), quercetin content was dropped by 24 % for OJB25 and 38 % for OJB50 group. Present study findings indicated that deposition of quercetin was enhanced when broiler birds were provided diet containing α-tocopherol and quercetin.

**Table 5:** Alpha tocopherol and quercetin content in broiler breast meat

Treatments	Alpha tocopherol contents (mg/kg meat)			Quercetin contents (mg/kg of meat)		
	Year 1	Year 2	Mean	Year 1	Year 2	Mean
T0	10.98 ± 0.21	10.42 ± 0.12	10.70 ± 0.17 g	-	-	-
T1	13.57 ± 0.55	12.83 ± 0.50	13.20 ± 0.37f	3.68 ± 0.22	3.57 ± 0.15	03.62 ± 0.13e
T2	17.70 ± 0.23	17.60 ± 0.35	17.65 ± 0.19ef	4.20 ± 0.24	4.13 ± 0.20	04.16 ± 0.17de
T3	24.13 ± 0.70	23.77 ± 0.57	23.95 ± 0.41de	14.16 ± 1.05	14.11 ± 1.12	14.13 ± 1.06b
T4	16.77 ± 0.43	16.57 ± 0.33	16.67 ± 0.24e	8.74 ± 0.54	8.67 ± 0.64	08.70 ± 0.63 cd
T5	24.35 ± 0.96	24.13 ± 0.67	24.24 ± 0.53d	9.12 ± 0.64	9.01 ± 0.75	09.06 ± 0.74c
T6	30.12 ± 0.39	29.67 ± 0.35	29.89 ± 0.25c	9.92 ± 0.51	9.87 ± 0.63	09.89 ± 0.72bc
T7	18.46 ± 1.17	18.02 ± 0.87	18.24 ± 0.66e	4.85 ± 0.13	4.74 ± 0.15	04.79 ± 0.14d
T8	31.97 ± 0.89	31.57 ± 0.82	31.77 ± 0.55b	14.73 ± 1.04	14.62 ± 1.03	14.67 ± 1.03ab
T9	38.77 ± 0.62	37.27 ± 0.64	38.02 ± 0.52a	16.38 ± 1.12	16.34 ± 1.07	16.36 ± 1.01a
Means	22.68 ± 1.57	22.18 ± 1.53		8.58 ± 0.95	8.51 ± 0.91	

Means sharing similar letters in a column do not differ significantly from one another (*p* 0.05)

T0 = control without antioxidants, T1 = 100 mg quercetin + 150 mg α-tocopherol/kg feed; T2 = 100 mg quercetin + 225 mg α-tocopherol/kg feed; T3 = 100 mg quercetin + 300 mg α-tocopherol/kg feed; T4 = 200 mg quercetin + 150 mg α-tocopherol/kg feed; T5 = 200 mg quercetin + 225 mg α-tocopherol/kg feed; T6 = 200 mg quercetin + 300 mg α-tocopherol/kg feed, T7 = 300 mg quercetin + 150 mg α-tocopherol/kg feed; T8 = 300 mg quercetin + 225 mg α-tocopherol/kg feed, T9 = 300 mg quercetin + 300 mg α-tocopherol/kg feed

### Alpha tocopherol contents

The findings (Table 5) regarding α-tocopherol content of breast meat of broiler birds elucidated significant variations due to treatments however, non-substantial behavior was noticed for years and interaction of treatments × years. In treatments, means explicated the lowest for α-tocopherol contents of breast meat in T0 (control) 10.98 ± 0.21 mg/kg meat whereas, the highest in T9 (birds fed on 300 mg quercetin & 300 mg of α-tocopherol/kg feed) 38.77 ± 0.62 mg/kg meat followed by T8 (birds fed on 300 mg quercetin & 225 mg of α-tocopherol/kg feed) 31.77 ± 0.55 mg/kg meat, T6 (birds fed on 200 mg quercetin and 300 mg of α-tocopherol/kg feed) 29.89 ± 0.25 mg/kg feed, respectively. The results of instant study are supported by [34] who indicated that α-tocopherol concentration of breast and thigh muscles of birds was enhanced by increasing the level of dietary α-tocopherol. They further stated that feed supplementation @200 or 400 IU of α-tocopherol is effective in retarding the oxidative degradation of lipids and cholesterol. Likewise, [35] also reported fortification feed with α-tocopherol increases the deposition of α-tocopherol in meat. One of the researchers groups, [36] listed that dietary supplementation of α-tocopherol acetate significantly amplified its level in pectoralis muscles indicating its deposition in tissues. They also stated that α-tocopherol level of muscles was not decreased with postmortem time. One of the scientists groups, [37] reported that vitamin E supplemented feed to the broiler birds increased vitamin E concentration in breast muscle.

### Fatty acid profile of broiler breast meat

The results (Table 6) elucidated that fatty acid composition of broiler meat affected significantly among treatments whilst years affected non-momentously. It has been observed 8 fatty acid in meat samples mainly palmitic acid (C16:0), stearic acid (18:0), oleic acid (18:1n-9) and linolieci acid (18:2n-9, 6). Nonetheless, α-linolenic acid (18:3) and arachidonic acid (20:4) are not differed substantially among treatments in both years. It is obvious from findings that the lowest amount of palmitic acid (16:0) recorded in T9 13.4 & 12.5 % followed by T8 and T6 as 14.3 & 13.2, 14.85 & 13.9 % whereas highest in T0 19.6 & 18.4 % in respective years. Likewise, minimum stearic acid (16:0) was also found in T9 7.14 & 7.02 % trailed by T8 and T6 7.56 & 7.01 and 7.96 & 7.04 % though, highest in T0 10.06 & 9.84 %. Furthermore, highest level of oleic acid (18:1) reported in T0 24.38 & 23.35 % followed by T2 and T1 as 24.16 & 23.05, 23.76 & 22.65 % however, lowest in T6 21.01 & 19.93 %. The results expounded lowest saturated fatty acid (SFA) in T9 21.69 % whereas the value of this trait in T8, T6 and T0 as 23.01, 23.93, 30.76 %. Similarly, polyunsaturated fatty acids (PUFA) including oleic, linoleic and linolenic acids in different groups varied from 9.88 to 13.41 % and 9.62 to 12.60 % in respective years (Table 7). Overall, fatty acid production decreased with increasing level of quercetin and α-tocopherol in a dose dependent manner that depicted their lipid lowering potential however, reduction in SFA fatty acids was more obvious than that of PUFA in broiler meat. The findings of instant study are in harmony with [38] they stated that dietary supplementation of quercetin @200 mg/kg feed to broilers affect fatty acid composition of pectoralis muscle of birds. They recorded oleic (18:1n-9, 33–35.2 %), palmitic (16:0, 26.1-27.9 %), linoleic (18:2n-6, 14.0-14.6 %) and stearic acid (18:0, 11.8–13.7 %) in pectoralis muscle, respectively and further elaborated that quercetin addition significantly (p < 0.05) diminishes palmitic, oleic and linoleic acid production. Likewise, [39] noticed that dietary administration of quercetin @1 % in rat affected fatty acid synthesis rate and diminished free fatty acids production. The mechanistic approach for reduction of SFA in broiler meat due to antioxidants supplementation is their potential to inhibit activity of 9-desaturase complex that converts SFA to MUFA One of the scientists groups [40], delineated that quercetin decreases the rate of fatty acid and triacylglycerol (TAG) synthesis in rats. One of the researchers groups [41], documented a declining trend in the production of SFA and MUFA in broilers breast meat through dietary supplementation of gallic acid in combination with linoleic acid. One of the scientists groups [42], specified that inclusion of flavonoid i.e. genistein and hesperidin in diet decreased SFA level in breast meat. They conducted a study in which one day old 360 birds were fed on control, G_5_ containing 5 mg of genistein, H_2_0 containing 20 mg hesperidin, GH5 having genistein & hesperidin @5 mg/kg, GH10 include genistein & hesperidin @10 mg/kg) GH20 having genistein & hesperidin @20 mg/kg) diets, respectively. The results delineated that GH20 group decreased fatty acid proportions of myristic and oleic acid with reduction of 30 and 8.7 %, respectively. Compared to control, genistein and hesperidin-supplemented group significantly diminished fatty acid proportion of myristic & stearic acid in dose-dependent manner. Furthermore, ratio of Ω-6 to Ω-3 fatty acid were also enhanced through genistein and hesperidin provision with maximum effect of 37 % in GH20 group. The changes induced by quercetin and α-tocopherol in current study showed similar pattern for reduction of fatty acid in muscles of the broiler birds.

**Table 6:** Fatty acids composition of broiler breast meat

Treatments	2013								2014							
	^a^14:0	^b^16:0	^c^16:1	^d^18:0	^e^18:1	^f^18:2	^g^18:3	^h^20:4	^a^14:0	^b^16:0	^c^16:1	^d^18:0	^e^18:1	^f^18:2	^g^18:3	^h^20:4
T0	1.10c	19.6a	1.65d	10.06a	24.38ab	11.61a	0.86ab	0.94b	1.01c	18.40a	1.50b	9.84a	23.35ab	10.98a	0.76c	0.86b
T1	1.19a	18.4ab	1.78c	9.78ab	23.76bc	11.14ab	0.81bc	0.96ab	0.99c	17.26ab	1.60a	9.12ab	22.65bc	10.28ab	0.81ab	0.91a
T2	0.99d	17.6b	1.67 cd	9.41b	24.16b	10.65b	0.84b	0.89c	1.14a	16.40b	1.52b	8.98b	23.05b	10.31ab	0.79b	0.87b
T3	1.20a	17.11bc	2.1a	8.98c	23.48c	9.96c	0.82bc	0.98a	1.16a	16.14c	1.51b	8.16c	22.18c	9.34bc	0.84a	0.82c
T4	1.17a	17.56b	1.84bc	9.11bc	24.64a	10.26bc	0.89a	0.94b	1.06bc	16.20bc	1.60a	8.64bc	23.71a	9.76b	0.80b	0.92a
T5	1.18a	16.8c	1.87b	8.63 cd	21.07e	9.61 cd	0.86ab	0.94b	1.08b	15.70 cd	1.55ab	8.11 cd	20.02e	9.10c	0.85a	0.89ab
T6	1.12b	14.85d	2.06a	7.96de	21.01e	8.56de	0.82bc	0.98a	1.04bc	13.90de	1.51b	7.04e	19.93f	8.09de	0.82ab	0.86b
T7	1.12b	16.3 cd	1.85bc	8.26d	22.79 cd	9.11d	0.78c	0.91bc	1.10b	15.23d	1.45c	7.86d	21.78 cd	9.01 cd	0.82ab	0.84bc
T8	1.15ab	14.3de	1.93ab	7.56e	21.45d	8.26e	0.87ab	0.91bc	1.08b	13.20e	1.56ab	7.01e	20.41de	8.21d	0.84a	0.91a
T9	1.15ab	13.4e	1.78c	7.14f	21.15de	8.08f	0.86ab	0.94b	1.12ab	12.50f	1.60a	7.02e	20.65d	8.01e	0.78bc	0.83c

Means sharing similar letters in a column do not differ significantly from one another (p 0.05)

^a^Myristic, ^b^Palmitic, ^c^Palmitoleic, ^d^stearic, ^e^Oleic, ^f^Linoleic, ^g^α-Linolenic, ^h^Arachidonic

T0 = control without antioxidants, T1 = 100 mg quercetin + 150 mg α-tocopherol/kg feed; T2 = 100 mg quercetin + 225 mg α-tocopherol/kg feed; T3 = 100 mg quercetin + 300 mg α-tocopherol/kg feed; T4 = 200 mg quercetin + 150 mg α-tocopherol/kg feed; T5 = 200 mg quercetin + 225 mg α-tocopherol/kg feed; T6 = 200 mg quercetin + 300 mg α-tocopherol/kg feed, T7 = 300 mg quercetin + 150 mg α-tocopherol/kg feed; T8 = 300 mg quercetin + 225 mg α-tocopherol/kg feed, T9 = 300 mg quercetin + 300 mg α-tocopherol/kg feed

**Table 7:** Effect of treatments on saturated, Monounsaturated and polyunsaturated fatty acid composition of broiler breast meat

Treatments	2013				2014			
	ΣSFA	ΣMUFA	ΣPUFA	PUFA: SFA	ΣSFA	ΣMUFA	ΣPUFA	PUFA: SFA
T0	30.76a	25.81b	13.41a	0.45	29.25a	24.55b	12.60a	0.43
T1	29.37ab	25.54bc	12.91ab	0.44	27.37b	24.25bc	12.10ab	0.44
T2	28.00b	23.12d	12.38b	0.44	26.52bc	24.87ab	11.97b	0.45
T3	27.29c	23.25 cd	11.76c	0.44	25.46 cd	23.69c	11.00c	0.43
T4	27.84bc	22.85de	12.09bc	0.43	25.90c	25.31a	11.48bc	0.44
T5	26.61 cd	21.87e	11.41c	0.43	24.89d	21.57e	10.84 cd	0.44
T6	23.93de	23.13d	10.36d	0.43	21.98e	21.44e	9.77e	0.44
T7	25.68d	24.64c	10.8 cd	0.42	24.19de	23.23 cd	10.67d	0.44
T8	23.01e	26.57a	10.04de	0.43	21.29ef	21.97de	9.96de	0.46
T9	21.69f	26.16ab	9.88e	0.45	20.64f	22.25d	9.62f	0.46

Means sharing similar letters in a column do not differ significantly from one another (*p* 0.05)

T0 = control without antioxidants, T1 = 100 mg quercetin + 150 mg α-tocopherol/kg feed; T2 = 100 mg quercetin + 225 mg α-tocopherol/kg feed; T3 = 100 mg quercetin + 300 mg α-tocopherol/kg feed; T4 = 200 mg quercetin + 150 mg α-tocopherol/kg feed; T5 = 200 mg quercetin + 225 mg α-tocopherol/kg feed; T6 = 200 mg quercetin + 300 mg α-tocopherol/kg feed, T7 = 300 mg quercetin + 150 mg α-tocopherol/kg feed; T8 = 300 mg quercetin + 225 mg α-tocopherol/kg feed, T9 = 300 mg quercetin + 300 mg α-tocopherol/kg feed

SFA = Saturated fatty acids

MUFA = Mono unsaturated fatty acids

PUFA = Poly unsaturated fatty acids

PUFA/SFA = Ratio of polyunsaturated fatty acids and saturated fatty acids

## Conclusion

The findings of instant exploration revealed that antioxidant potential of broilers breast meat can be improved by the supplementing quercetin in combination with alpha tocopherol. The supplementation of birds feed with 300 mg/kg feed dietary level of quercetin in combination with alpha tocopherol improved the growth performance by increasing gain in weight and lowering FCR of birds. Accordingly, the antioxidant potential was also improved through supplementation of quercetin and alpha tocopherol that increases the stability of lipid against oxidation in meat. The results further elaborates that fatty acid production decreased with increasing level of quercetin and α-tocopherol in dose dependent manner however, reduction in SFA fatty acids was more pronounced than that of PUFA content of broiler breast meat.

## Materials and methods

The current study was conducted at the National Institute of Food Science and Technology (NIFSAT) and Nutrition Research Center, University of Agriculture, Faisalabad, Pakistan. In present research, the influence of dietary quercetin and α-tocopherol via feed supplementation in broiler was explored to enhance the antioxidant potential and lipid stability of broiler breast meat. The instant study was approved by the National Institute of Food Science and Technology review board. Materials and protocols followed to carry out this study are described herein.

### Procurement of raw materials

All reagents and chemicals required for the instant exploration were purchased from Sigma Aldrich (Tokyo, Japan) and Merck (Merck KGaA, Darmstadt, Germany). The quercetin was acquired from Shaanxi Jintai Biological Engineering Co. Ltd. China. Alongside, 300 one day old broiler chicks (50 ± 5 g body weight) were procured from Jadeed Chicks Pvt. Ltd. Faisalabad, Pakistan. The study was conducted in duplicate to enhance the authenticity of results.

### Diet, study plan and management of birds

In the current study, 300 one day old broiler chicks were used as experimental animals for the production of functional broiler meat. The animals were treated by following guidelines of the ethical committee as approved by the university. For the intention, they were weighed individually and divided randomly into 10 groups each consisting of 30 birds reared for a period of six weeks (Table 8). The composition of the control feed provided to the birds is mentioned in Table 9. Prior to research, the research area and all pens were thoroughly cleaned. For disinfection, bromosept and formalin aqueous solution with 1:12 ratio was used in the experimental premises. Likewise, 2–3 in. thick layer of saw dust was spread in each pen as a litter to keep the bed dry and soft. Moreover, all drinkers and feeders were thoroughly washed and disinfected during the course of research trial. All the pens were tagged with respective treatments and replication numbers. The temperature of the experimental room was maintained at 95 ± 2 °F during the first week of trial followed by a decrease of 5 °F till reached to 75 ± 2 °F. The light and proper ventilation were also maintained in the experimental room. The experimental birds were provided free access to feed and fresh water.

**Table 8:** Broilers feed supplementation plan

Treatment	Description
T_0_	Control diet without antioxidants
T_1_	100 mg quercetin + 150 mg α-tocopherol/kg feed
T_2_	100 mg quercetin + 225 mg α-tocopherol/kg feed
T_3_	100 mg quercetin + 300 mg α-tocopherol/kg feed
T_4_	200 mg quercetin + 150 mg α-tocopherol/kg feed
T_5_	200 mg quercetin + 225 mg α-tocopherol/kg feed
T_6_	200 mg quercetin + 300 mg α-tocopherol/kg feed
T_7_	300 mg quercetin + 150 mg α-tocopherol/kg feed
T_8_	300 mg quercetin + 225 mg α-tocopherol/kg feed
T_9_	300 mg quercetin + 300 mg α-tocopherol/kg feed

**Table 9:** Composition of basal feed provided to the birds

Ingredients	Quantity (g/kg feed)
Corn	490
Rice broken	20.7
Rice polishing	56.0
Cotton seed meal	22.0
Canola meal	20.0
Corn gluten 60 %	23.0
Sunflower meal	124.0
soybean meal	150.0
Fish meal	66.0
L-lysine	1.50
DL-methionine	00.8
Dicalcium phosphate	12.0
Limestone	12.0
Premix	02.0
Nutrient composition (calculated)	
Metabolized energy (Kcal /kg)	2934
Crude protein (%)	21.03
Lysine (%)	1.10
Methionine (%)	0.52

### Vaccination schedule of chicks

The glucose solution (50 g/5 L) was given to the chicks after 2 h of distribution in pens for waste removal. On 2nd day, cotrim-50 solution (1 g/5 L of water) was administrated to chicks as an antibacterial agent. The chicks were vaccinated for new castle disease (N.D) and infectious bronchitis (I.B) at 3rd and 4th day for the prevention from respective diseases. Afterwards, on 10th and 18th day, birds were vaccinated against gamboro disease by respective vaccine. Lastly, on 24th day, birds were vaccinated against new castle disease with Lasuta vaccine.

### Slaughtering, samples collection and preparation

The experimental birds were reared up to six weeks. For acclimatization, the chicks were fed on control diet during the first two weeks of study. Afterwards, they were fed on diet supplemented with quercetin and α-tocopherol as per treatment plan. At termination, the broiler birds were slaughtered by adopting Halal Islamic Ethical Guidelines. After slaughtering, breast muscles of broilers were separated, deboned, wrapped with aluminum foil and packed in polythene zip lock bags followed by storage at −18 °C for analysis. For sample preparation, 5 g meat sample was taken in 50 mL capped polypropylene tube and homogenized by using phosphate buffer and glycerol (20 %) at pH 7.4 through homogenizer. The tubes were placed in ice cold water to prevent the oxidation of muscle samples. Afterwards, filtration of samples was carried out to remove connective tissues.

### Growth parameters

The birds were weighed on weekly basis to calculate the weight gain. A measured quantity of feed was provided to chicks during each week for exploring the influence of added antioxidants on feed intake of broilers. Additionally, the mortality rate of the broiler birds during the course of research trial was also calculated. At the termination of research trial, feed conversion ratio of broilers (FCR) was calculated by dividing the feed consumed by weight gained in the respective week using the following expression;

$$ \mathrm{F}\mathrm{C}\mathrm{R} = \mathrm{F}\mathrm{eed}\ \mathrm{consumed}\ \mathrm{b}\mathrm{y}\ \mathrm{the}\ \mathrm{b}\mathrm{ird}\ \mathrm{in}\mathrm{oneweek}/\mathrm{Weight}\ \mathrm{gain}\ \mathrm{b}\mathrm{y}\ \mathrm{the}\ \mathrm{b}\mathrm{ird}\ \mathrm{in}\ \mathrm{respective}\ \mathrm{week} $$

### Antioxidant potential of meat

All the methods used to carry out the instant research were officially approved to carry out analysis. Antioxidant potential of breast meat samples of birds was estimated by using respective analytical methods;

### Total phenolic contents

The total phenolic contents (TPC) in breast broiler meat samples were determined by adopting the procedure as described by [43]. The homogenized meat sample (100 μL) was mixed with 500 μL (95 % ethanol), distilled water (2.5 mL) and 250 μL of 50 % Folin-Ciocalteu reagent. After 5 min, 250 μL of 5 % Na2CO3 was added to the resultant mixture, vortex and placed in the dark room for 1 h. Afterwards, absorbance of samples was recorded at 725 nm through UV/Visible Spectrophotometer (CECIL-CE7200) against control. The total phenolic contents of meat samples were estimated as gallic acid equivalent (mg gallic acid/g).

### Free radical scavenging activity

The free radical scavenging activity i.e. DPPH (1,1-diphenyl-2-picrylhydrazyl) of breast meat samples was measured using the protocol of [44]. Purposely, 1 mL of DPPH solution was added to 4 mL of sample followed by incubation for 30 min at room temperature. The absorbance was measured at 520 nm using UV/Visible Spectrophotometer. The DPPH free radical scavenging activity was calculated by the below mentioned equation;

$$ \mathrm{Inhibition}\left(\%\right) = 100 \times \left({\mathrm{A}}_{\mathrm{blank}}\ \hbox{-}\ {\mathrm{A}}_{\mathrm{sample}}/{\mathrm{A}}_{\mathrm{blank}}\right) $$

A_blank_ = absorbance of blank sample (t = 0 min)

A_sample_ = absorbance of tested solution (t = 15 min)

### Ferric reducing antioxidant power

The ferric reducing antioxidant power of breast broiler meat samples was estimated by following the procedure of [45]. The homogenized sample (200 μL) was mixed with 500 μL sodium phosphate buffer (0.2 M, pH 6.6) and 500 μL potassium ferric cyanide (1 %) followed by incubation at 50 °C in a water bath for 20 min. After cooling, sample was mixed with 2.5 mL (10 % TCA), distilled water (1.25 mL) and 0.25 mL (0.1 % ferric chloride) for 10 min. The absorbance was measured at 700 nm. During the analysis, an increase in the absorbance of the reaction mixture indicated the higher reducing power of the samples.

### Lipid stability by thiobarbituric acid reactive substances assay

The oxidative stability of broiler breast meat samples was measured by using thiobarbituric acid reactive substances (TBARS) according to the guidelines of [46]. In this context, 5 g of ground broiler meat samples were weighed in a 50 mL test tube and homogenized with 50 μL of butylated hydroxytoluene (7.2 %) and 15 mL of deionized distilled water using a homogenizer for 15 s. One mL of meat homogenate was transferred to a disposable test tube (13 × 100 mm) and 2 mL of TBA/trichloroacetic acid (TCA; 15 mM TBA/15 % TCA) solution was added. The mixture was vortex and incubated in a boiling water bath for 15 min to develop color. Afterwards, samples were cooled in ice water for 10 min, vortex again and centrifuged for 15 min at 2000 × *g* at 4 °C. The absorbance of the resulting supernatant solution was determined at 531 nm against a blank containing 1 mL of deionized distilled water and 2 mL of TBA/TCA solution. The amounts of TBARS were expressed as milligrams of malondialdehyde (MDA) per kilogram of meat. The amounts of TBARS were calculated by using formula as described below

$$ \mathrm{n}\hbox{-} \mathrm{mole}\ \mathrm{of}\ \mathrm{malondialdehydes} = \frac{\left(\mathrm{Sample}\ \mathrm{absorbance}\ \hbox{--}\ \mathrm{Blank}\right) \times \mathrm{Total}\ \mathrm{sample}\ \mathrm{volume}}{0.000156 \times 1000} $$

### Quantification of antioxidants

#### Quercetin contents

The quercetin content of breast meat samples was estimated through HPLC by following the protocol of [47]. Purposely, 2 g meat was mixed with 50 mL of 70 % v/v methanol/water. The mixture was homogenized for 5 min with a blender. The solution was filtered through a Whatman No. 4 filter paper under reduced pressure. The residue was extracted again with 50 mL of 70 % v/v methanol/water for 5 min followed by filtration through a Whatman No. 4 filter paper and in the resulting filtrate, methanol was added to make a volume of 100 mL. For HPLC analysis, the solution was filtered through a 0.45 mL of nylon filter disc prior to analysis. Afterwards, 1 mL of 1000 mg/mL sorbic acid solution (internal standard) was added and the total volume (25 mL) was made with methanol. The solution was filtered through a 0.45 mm nylon filter before analysis. The mobile phase comprised of 0.1 % formic acid in water and methanol with gradient of 20:80 %. The stock and working standards of quercetin were prepared in methanol. The analyses were performed with an Agilent series 1100 quaternary solvent delivery system with cooled autosampler (4 °C) and photodiode array detector (Agilent, Waldron Germany) connected to a thermo electron ion trap mass spectrometer operating in negative ion electrospray mode (Thermo Electron, San Jose, USA). The column was maintained at 30 °C and mobile phase consisted of (A) 0.1 % formic acid in water and (B) 0.1 % formic acid in methanol with the following gradient; 20:80 % (0–10 min), 80 % B (10–20 min), 80:20 % (20– 20.5 min), 20 % B (20.5–25 min) and flow rate of 0.2 mL/min. The HPLC column was fitted with a C_18_ column maintained at 30 °C and a mobile phase of (A) 0.1 % formic acid in water and (B) 0.1 % formic acid in acetonitrile with the following gradient: 5 % B (0–5 min), 5:80 % (5–25 min), 80 % B (25–30 min), 80–5 % (30–31 min), 5 % B (31–35 min) with a flow rate of 0.2 mL/min. All chromatograms were monitored at a wavelength of 280, 346, 364 and 370 nm.

#### Alpha tocopherol content

The α-tocopherol content of meat samples was measured by the protocol of [48]. The homogenized meat sample (500 μL) was taken in a test tube followed by the addition of 1.5 mL of urea (6 M) to dissolve the meat tissue. Later, 0.5 mL of ascorbic acid (5 %) was added in the reaction mixture to prevent the oxidation of α-tocopherol in the meat samples along with 1 mL of 6 M urea. The tubes were flushed with N_2_ and resultant mixture was vortex for 12 min to extract the tocopherol components of samples. Next, 1 mL of 0.1 M sodium dodecyl sulfate (SDS) solution was added and vortex for 1 min to disintegrate the meat tissue. For deproteination and release of α-tocopherol, 4 mL of ethyl alcohol containing 1 % pyrogallol was added in the resulting mixture. Thereafter, petroleum ether (10 mL) was added and the resultant mixture was centrifuged at 5000 × *g* for 5 min to facilitate the separation of phases. The solvent layer containing α-tocopherol was separated in the vial and the pooled solvent was evaporated under nitrogen. Alpha tocopherol content was dissolved in the mobile phase (100 % methanol) and then filtered through 0.45 μm microfilter, centrifuged at 5000 × *g* for 5 min to collect the filtrate and stored for HPLC analysis. The mobile phase comprised of methyl alcohol (HPLC grade); 100 % methanol was prepared by filtering through typhlon filter assembly and then adjusted according to requirements of HPLC. The standard of α-tocopherol was prepared by using Sigma Aldrich packed standard 1 mg/mL of α-tocopherol as stock solution from which further dilutions (10, 20, 50 and 100 μg/mL of solutions) were prepared. The α-tocopherol was extracted and quantified by using HPLC (PerkinElmer, Series 200, USA) chromatographic system at 290 nm with UV-Visible detector. The HPLC chromatograms were obtained through C_18_ column (250 mm × 4.6 mm, 5.0 μm), system controller SCL-10 A, water pump (LC-10 AT) and flow controller valve (FCV-10 AL) with a mobile phase of 100 % methanol at a flow rate of 1 mL/min.

#### Fatty acids profile

The fatty acid composition of breast meat samples was estimated by adopting the protocol of [49]. Accordingly, 2 g meat samples were weighed into a test tube with 20 mL of Folch solution (10 volumes, chloroform: methanol = 2:1, wt/vol) and homogenized using a polytron for 10 s. Moreover, 24 μL of butylated hydroxyanisole (BHA, 10 % dissolved in 98 % ethanol) was added to each sample prior to homogenization. The homogenate was filtered through whatman no.1 filter paper into a 100 mL graduated cylinder and ¼ volume (on the basis of Folch solution volume) of 0.88 % NaCl solution was added. Afterwards, the cylinder was capped with a glass stopper and the filtrate was mixed well. The cylinder was washed twice with 10 mL of Folch solution (3:47:48/CHCl_3_:CH_3_OH:H_2_O) and the contents were stored up to 6 h until aqueous and organic layers were clearly separated. After separation, upper layer containing methanol was siphoned and 0.5 mL of lower layer (chloroform layer) was moved to a glass scintillation vial and dried at 70 °C under nitrogen for 2–3 min. Moreover, 1 mL of BF_3_ in methanol was added as methylating agent to cut ester bond to form fatty acids methyl esters and then heated for 50 min followed by cooling at room temperature. Later, 3 mL of hexane and 5 mL of distilled water were added, mixed thoroughly and left overnight for phase separation. The top (hexane) layer, containing methylated fatty acids was used for gas chromatographic analysis. The fatty acid compositional profiling was performed by using Gas Chromatograph (HP 6890) equipped with an auto sampler and flame ionization detector. A capillary column (HP-5; 0.25 mm i.d., 30 m, 0.25-μm film thickness) was used to inject samples (1 μL) into the capillary column. The oven temperature conditions (180 °C for 2.5 min, increased to 230 °C at 2.5 C/min, then held at 230 °C for 7.5 min) were maintained. The temperatures of the inlet and detector were fixed at 280 °C. The helium was used as a carrier gas and a constant column flow of 1.1 mL/min was used. The flame ionization detector air, hydrogen (H_2_) and helium flows were 350, 35, and 43 mL/min, respectively. The identification of fatty acids was accomplished by comparing mass spectral of fatty acids against their standards. The results of the fatty acid were reported as percentage composition of total lipids and peak area was used to calculate fatty acid composition of samples.

#### Statistical analysis

The resultant data were analyzed through completely randomized design (CRD) by using package of statistical (Statistic 8.1). Moreover, Analysis of variance (ANOVA) was performed to measure the level of significance [50].
